# Bacterial Vaginosis‐Associated *Prevotella timonensis* Enhances Dendritic Cell–T Cell Clustering and Subsequent T Cell Proliferation

**DOI:** 10.1002/eji.70051

**Published:** 2025-09-03

**Authors:** Marleen Y. van Smoorenburg, Julia L. Nerwinska, John L. van Hamme, Ester B. M. Remmerswaal, Celia Segui‐Perez, Karin Strijbis, Teunis B. H. Geijtenbeek

**Affiliations:** ^1^ Department of Experimental Immunology Amsterdam UMC, Location University of Amsterdam Amsterdam The Netherlands; ^2^ Amsterdam institute for Immunology and Infectious Diseases Amsterdam The Netherlands; ^3^ Department of Biomolecular Health Sciences, Division of Infectious Diseases and Immunology, Faculty of Veterinary Medicine University of Utrecht Utrecht The Netherlands

**Keywords:** adhesion, bacterial infections, CD4 T cells, cell–cell clustering, cellular proliferation, dendritic cells (DCs), immune regulation, *Prevotella timonensis*, vaginal dysbiosis, vaginal microbiome

## Abstract

Dysbiosis of the vaginal microbiome is associated with increased inflammation in the female genital tract. Microbiota associated with bacterial vaginosis (BV), such as *Gardnerella vaginalis*, *Megasphaera elsdenii*, and *Prevotella timonensis*, replace the health‐associated bacterium *Lactobacillus crispatus* and cause inflammation affecting mucosal integrity and immunity. However, it remains unclear how these BV‐associated bacteria modulate immune cells and enhance inflammation. Here, we investigated whether BV‐associated bacteria directly affected dendritic cell (DC) function. Notably, *P. timonensis* but not *M. elsdenii* induced cell–cell clustering between monocytic cell lines and, importantly, between primary DCs and primary CD4 T cells. Our data indicate that this increased clustering is independent of LFA‐1. Moreover, *P. timonensis* enhanced DC‐mediated CD4 T cell proliferation. Altogether, these results suggest that *P. timonensis*‐induced cell–cell clustering contributes to the elevated mucosal inflammation observed during bacterial vaginosis.

## Graphical abstract

*Prevotella timonensis*, a bacterium present during vaginal dysbiosis, increases cell–cell clustering between primary DCs and CD4 T cells and subsequently enhances CD4 T cell proliferation. These results provide insight into how microbiota directly affect the immunological function of DCs, thereby modulating inflammation. Created in BioRender. Geijtenbeek, T. (2025) https://BioRender.com/t02mfss.

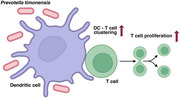

## 1 Introduction

Bacterial vaginosis (BV) is a condition prevalent among women of all ages, including reproductive‐aged women, and characterized by an imbalance of vaginal microbiota in the female genital tract [1]. BV is associated with elevated mucosal inflammation, which has been implicated as a mechanism responsible for BV‐associated afflictions, including decreased fertility and adverse pregnancy outcomes, such as preterm birth [1, 2, 3]. Dysbiosis of the vaginal microbiome is characterized by an increase in diversity of predominantly anaerobic bacteria and a loss of health‐associated *Lactobacillus* spp. [1, 2, 4, 5]. *Lactobacillus* spp. are important in establishing immune tolerance, whereas the shift to anaerobic bacteria such as *Gardnerella vaginalis*, *Megasphaera elsdenii*, and *Prevotella timonensis* drives mucosal inflammation [4, 6]. However, the underlying mechanisms and roles of different bacterial species in inflammation remain unclear.

Dendritic cells (DCs) survey the vaginal mucosa and play a pivotal role in the mucosal immune response against these BV‐associated bacteria [7]. *P. timonensis* and *M. elsdenii* trigger pattern recognition receptors on DCs, leading to strong DC maturation and production of proinflammatory cytokines [8, 9], which might underlie the observed mucosal inflammation. Interestingly, we have recently shown that *P. timonensis* enhances HIV‐1 uptake by different DC subsets, whereas other BV‐associated bacteria do not [9, 10]. This suggests that specific bacteria directly affect DC function in HIV‐1 infection in unique ways. Here, we investigated whether BV‐associated bacteria directly affect the immunological function of DCs, thereby modulating inflammation.

Pathogen recognition induces DC maturation and migration to lymph nodes, where DCs form clusters with T cells for antigen presentation, leading to T cell activation and proliferation [11], thereby initiating adaptive immunity. Hence, we assessed the ability of these BV‐associated bacteria to affect DC clustering and T cell activation. We have identified a novel mechanism of immune dysregulation by *P. timonensis*, which strongly increased clustering of DCs with primary CD4 T cells and subsequently enhanced CD4 T cell proliferation.

## 2 Results and Discussion

### 2.1 *Prevotella timonensis* Enhances DC–T Cell Clustering

**FIGURE 1:**
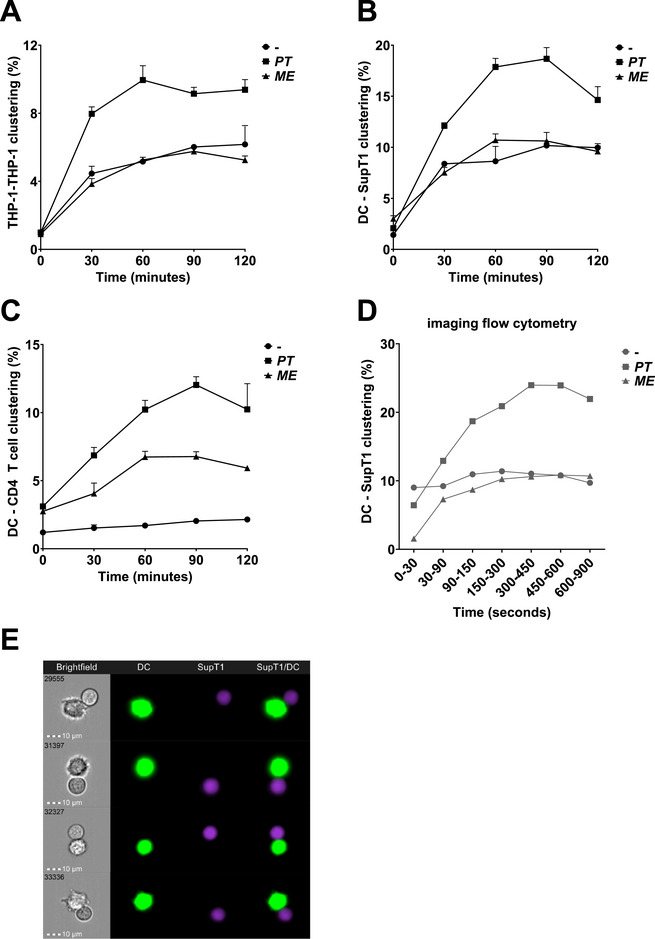
*Prevotella timonensis* enhances THP‐1 cell–cell clustering and DC–T cell clustering. THP‐1 cells (A) or DCs (B‐E) were fluorescently labelled with Carboxyfluorescein Succinimidyl Ester (CFSE; 1:5000) and stimulated with UV‐inactivated bacteria *P. timonensis* (*PT*) or *Megasphaera elsdenii* (*ME*) for 16 h (MOI 10). CFSE‐labelled THP‐1 cells (A) or DCs (B, C) were co‐cultured with dihydroethidium (HE; 1:1000)‐labelled THP‐1 cells (A), SupT1 cells (B) or CD4 T cells (C) for 2 h in a 1:1 ratio. Clustering was measured every 30 min by flow cytometry. Live cells were gated based on FSC‐A versus SSC‐A, and HE/CFSE double‐positive events (Q2) were considered clustered cells. (A–C) Percentage of cell–cell clustering depicted over time. Experiments were measured in triplicate and repeated independently three times. One representative is shown, and symbols represent different conditions depicted as mean +SD. *P. timonensis*‐exposed conditions reach maximal clustering with SupT1 cells (B) between [14.1% and 18.7%] or with CD4 T cells (C) between [9.6% and 13.3%] with different DC donors. (D, E) CFSE‐labelled DCs were co‐cultured with CellTrace Violet (CTV)‐labelled SupT1 cells in a 1:1 ratio, and clustering was measured over time from 0 to 900 s by imaging flow cytometry. Cell–cell clustering (T + DC gate) was depicted as a percentage of all CFSE‐positive cells. Experiments were repeated independently three times; one representative is shown. (E) Four representative images depicting cell–cell clustering from one donor in a *P. timonensis*‐exposed condition within the T + DC gate in the time gate 450–600 s; showing respectively the brightfield image, signal in CFSE (DC) fluorescent channel, signal in CTV (SupT1) fluorescent channel, and overlay of signals in CFSE + CTV fluorescent channels.

We investigated whether vaginal dysbiosis‐associated bacteria affect cell–cell clustering, which is important for the induction of immune responses. CFSE‐labelled monocytic THP‐1 cells were treated with UV‐inactivated bacteria *P. timonensis* or *M. elsdenii* for 16 h, co‐cultured with HE‐labelled THP‐1 cells for 2 h, and cell–cell clustering was determined by flow cytometry (Figure S1). Notably, *P. timonensis* enhanced clustering of CFSE‐labelled THP‐1 cells with HE‐labelled THP‐1 cells compared with untreated cells (Figure 1A; Figure S1). Enhanced cell–cell clustering following *P. timonensis* exposure was observed within 30 min of co‐culture and remained stable over time (Figure 1A). Excluding doublets resulted in a loss of the observed clustering, confirming that the clustering is mediated via cell–cell contact (Figure S1). On the contrary, clustering was not affected by *M. elsdenii*. These data demonstrate that cell–cell clustering is enhanced in the presence of *P. timonensis*.

DC clustering with T cells is a crucial step in the induction of T cell activation and proliferation [11]. We therefore investigated whether *P. timonensis* enhanced clustering of primary DCs with T cells. CFSE‐labelled DCs were stimulated for 16 h with UV‐inactivated *P. timonensis* or *M. elsdenii*, and subsequently co‐cultured with the HE‐labelled T cell line SupT1 for 2 h. In all conditions, DC clustering with SupT1 cells increased over time (Figure 1B). Notably, exposure to *P. timonensis* but not *M. elsdenii* increased clustering of DCs with SupT1 cells compared with untreated DCs. Moreover, *P. timonensis* enhanced DC clustering with primary resting CD4 T cells to a higher extent than observed with *M. elsdenii* (Figure 1C). Whereas without any activation, DC clustering with CD4 T cells was low. To demonstrate actual cell–cell contact, we performed additional imaging flow cytometry analyses measuring cell–cell clustering of CFSE‐labelled DCs with CTV‐labelled SupT1 cells over time (Figure S1). *P. timonensis*, but not *M. elsdenii*, enhanced clustering of DCs with SupT1 cells over time (Figure 1D,E; Figure S1). Altogether, our data strongly suggest that *P. timonensis* enhances clustering of DCs with primary T cells and T cell lines. Therefore, we next investigated whether exposure to *P. timonensis* affected the adhesion molecules involved in DC–T cell clustering.

### 2.2 LFA‐1 Activation by DCs and Slightly Increased ICAM‐1 Binding by *P. timonensis*

**FIGURE 2:**
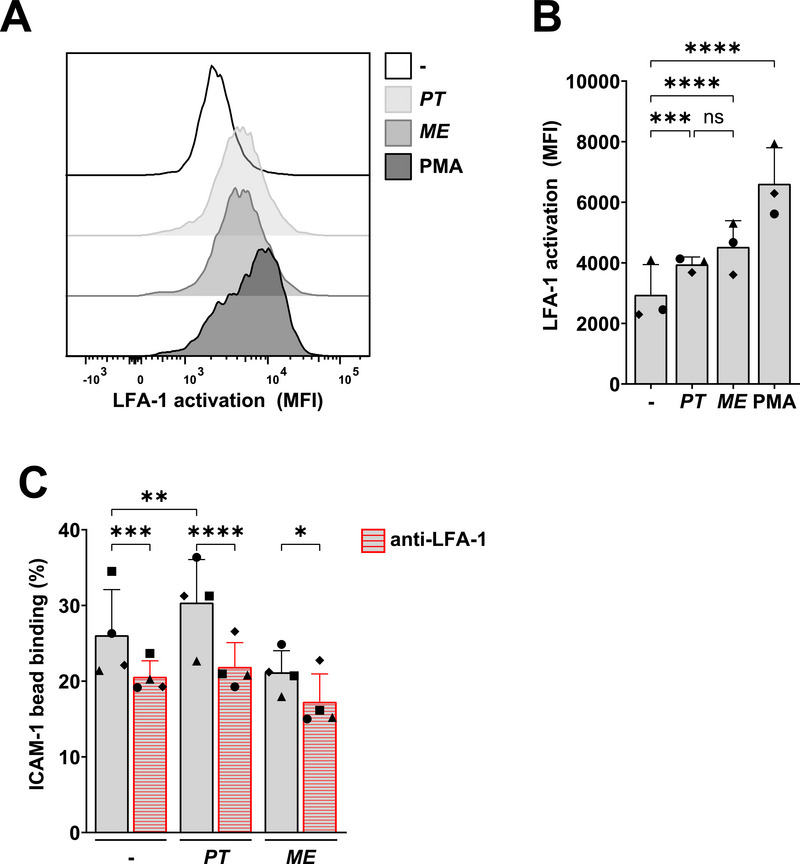
LFA‐1 activation by DCs and slightly increased ICAM‐1 binding by *P. timonensis*. DCs were stimulated with UV‐inactivated bacteria *P. timonensis* or *M. elsdenii* for 16 h (MOI 10). (A, B) DCs were pretreated with integrin stimulus phorbol 12‐myristate 13‐acetate (PMA, 100 ng/mL) and stained for activation of the LFA‐1 integrin receptor with mAb clone m24. Representative histogram of LFA‐1 activation for one donor (A) or combined flow cytometry data by geometric mean of the fluorescent intensity (MFI) (B) is depicted. C. DCs were pretreated with an LFA‐1 blocking antibody (40 µg/mL) for 30 min and subsequently exposed to fluorescent ICAM‐1 beads for 30 min. Bead binding was assessed by flow cytometry and depicted as a percentage of live cells with ICAM‐1 beads bound. Symbols represent independent donors, bars represent mean + SD. Experiments were performed for three (A, B) or four (C) different donors measured in duplicate (C) or triplicate (A, B). Statistical analysis was performed using a two‐way ANOVA with Tukey's multiple comparisons test. ****p < 0.0001, ***p < 0.001, **p < 0.01, *p < 0.05.

Interaction of the β2‐integrin LFA‐1 with ICAM‐1 is important for cellular contact between immune cells, and essential in the formation of immunological synapses, allowing for sustained DC clustering with T cells [12, 13]. Monocyte‐derived DCs express the β2 integrin LFA‐1 [12], and the high‐affinity (extended‐open) conformation of LFA‐1 can be detected with the mAb clone m24. DCs were stained with m24 and measured by flow cytometry. The positive control phorbol 12‐myristate 13‐acetate (PMA) induced high levels of m24 staining, indicating activation of LFA‐1 on DCs. Similarly, both *P. timonensis* and *M. elsdenii* induced exposure of the m24 epitope (Figure 2A,B), which suggests that both bacteria induce LFA‐1 activation to a comparable extent.

Next, we assessed ICAM‐1 interactions with DCs using the ICAM‐1 fluorescent bead binding assay [14]. Exposure to *P. timonensis* induced a significant but minor increase in ICAM‐1 binding to DCs compared with untreated DCs, while *M. elsdenii* did not enhance ICAM‐1 binding (Figure 2C). The blocking antibody against LFA‐1 decreased the binding of ICAM‐1 to DCs in both bacteria‐exposed and untreated conditions. Altogether, these data suggest that both bacteria induce the extended‐open conformation of LFA‐1, probably due to DC maturation. Moreover, *P. timonensis* exposure induced a slight increase of ICAM‐1 binding to DCs in contrast to *M. elsdenii*, which does not seem to explain the strong *P. timonensis*‐induced induction of clustering observed between DCs and T cells. These data therefore indicate that the *P. timonensis*‐induced clustering is independent of LFA‐1. As complexes of ICAM‐1 with LFA‐1 stabilize DC–T cell interactions and sustain T cell activation [15], we investigated whether *P. timonensis* would have additional downstream effects on T cells.

### 2.3 *Prevotella timonensis* Enhances DC‐Induced T Cell Proliferation

**FIGURE 3:**
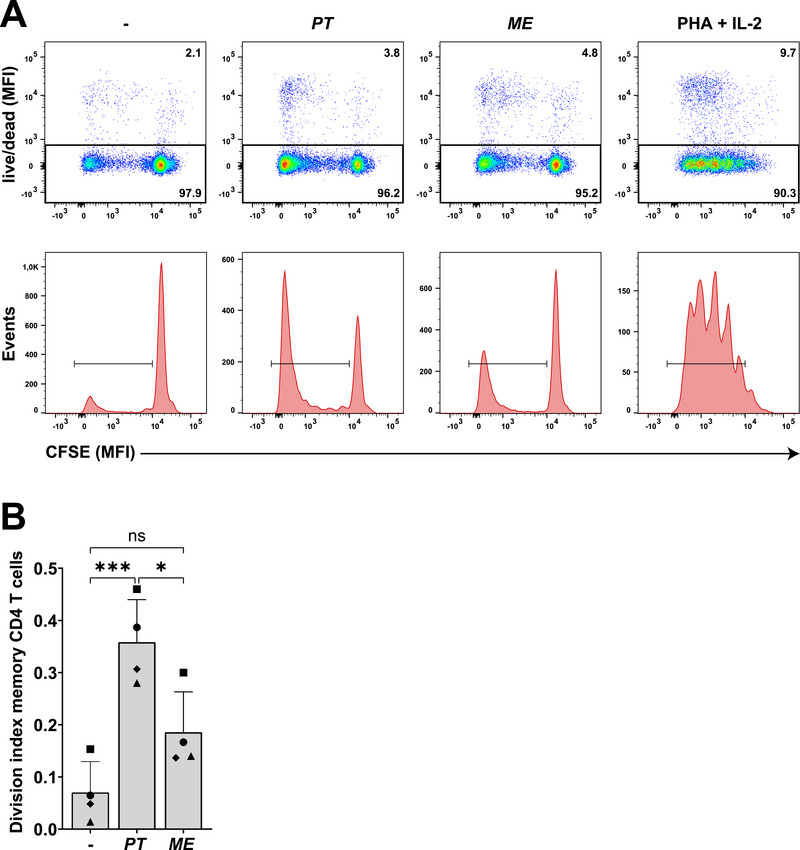
*P. timonensis* enhances DC‐induced T cell proliferation. DCs were stimulated with UV‐inactivated bacteria *P. timonensis* or *M. elsdenii*, for 16 h (MOI 10) and co‐cultured with CFSE‐labelled (1:5000) memory CD4 T cells (CD45RO) in a 1:4 ratio for five days. Stimulation with phytohemagglutinin (PHA, 1 µg/mL) and interleukin‐2 (IL‐2, 50 U/mL) was used as a positive control. (A) T cell proliferation was determined by loss of CFSE intensity by flow cytometry. Cells staining positive for the live–dead marker (Fixable Viability Dye eFluor 780) were excluded from analysis (% upper right). Subsequently, live cells (% lower gate) were depicted in a histogram and gated to determine the amount of T cell proliferation. (B) The division index of memory CD4 T cells was determined using the proliferation tool in FlowJo, and depicts the total number of cell divisions/the total number of viable, CFSE‐labelled cells at the start of the culture. Symbols represent independent donors, bars represent mean +SD. Experiments were performed for four different donors and measured in triplicate. Statistical analysis was performed using a two‐way ANOVA with Tukey's multiple comparisons test. ***p < 0.001, *p < 0.05.

We examined whether the enhanced cell–cell clustering induced by *P. timonensis* affects T cell proliferation. DCs were stimulated for 16 h with UV‐inactivated *P. timonensis* or *M. elsdenii*, subsequently co‐cultured with allogeneic CFSE‐labelled memory CD4 T cells for five days, and proliferation was assessed by flow cytometry. T cell proliferation in this allogeneic co‐culture reflects an alloresponse triggered by recognition of non‐self MHC molecules on moDCs by T cell receptors. Exposure to PHA and IL‐2, used as a positive control for direct DC‐independent T cell proliferation, induced T cell proliferation as determined by loss of CFSE due to cell divisions (Figure 3A). Notably, DCs exposed to *P. timonensis* enhanced T cell proliferation in comparison to T cells co‐cultured with *M. elsdenii*‐exposed DCs, and untreated DCs assessed by the division index (Figure 3A,B). *M. elsdenii* exposure also showed a trend toward enhanced proliferation, but the levels were lower than those observed for *P. timonensis*, in line with the observed clustering. As both bacteria induce comparable DC maturation and cytokine responses [8, 9], these data indicate that the enhanced clustering induced by *P. timonensis* strengthens DC–T cell interactions, resulting in subsequent T cell proliferation either by enhanced MHC–T cell receptor interactions or enhanced co‐stimulation.

## 3 Concluding Remarks

In conclusion, we have identified another level of immune dysregulation by *P. timonensis*, which directly impacts the function of DCs in adaptive immunity via enhanced clustering with CD4 T cells and subsequent increased CD4 T cell proliferation. Furthermore, our data suggest that increased clustering induced by *P. timonensis* is not dependent on TLR4 signaling, as both *P. timonensis* and *M. elsdenii* activate TLR4, inducing comparable DC activation and cytokines [8, 16], but solely *P. timonensis* enhances clustering. Our data suggest that the induced clustering by *P. timonensis* is independent of ICAM‐1/LFA‐1 interactions. Additional research needs to be done to investigate other adhesion pathways, such as CD2/CD58 (LFA‐3), or signaling lymphocytic activation molecule family (SLAMF) pathways, such as SLAMF1 (CD150), or SLAMF6. Our data suggest that the *P. timonensis*‐induced clustering leads to higher T cell proliferation by DCs, which might result in increased inflammation. Furthermore, our data with a monocytic cell line suggest *P. timonensis*‐induced clustering could also affect the function of other innate cells, such as monocytes and macrophages. We have recently shown that specifically *P. timonensis* strongly induces HIV‐1 uptake by Langerhans cells and DCs [9, 10]. This *P. timonensis*‐induced viral uptake led to enhanced transmission to susceptible target cells [9, 10]. HIV‐1 acquires host membrane proteins, such as ICAM‐1, during budding of infected cells [17], and it is possible that the enhanced DC–T cell clustering is also related to increased HIV‐1 uptake via these host membrane proteins, suggesting that there may be a similar mechanism involved.

Altogether, the immune cell–cell clustering induced by *P. timonensis* could contribute to the enhanced genital tract inflammation that is observed during bacterial vaginosis. We show that microbiota directly affect adhesion and cell–cell interactions, which could also be of importance in other *Prevotella*‐rich mucosal areas, such as the intestinal tract, where *Prevotella* spp. have been linked to chronic mucosal inflammation [18, 19, 20]. Identifying the underlying mechanisms of *Prevotella* effects on immune cells will facilitate the design of therapeutic approaches to ultimately reduce bacterial vaginosis and mucosal inflammation.

## 4 Data Limitations and Perspectives

Here, we focused on the induction of cell–cell clustering induced by *P. timonensis* in primary DCs as well as a monocytic cell line. We compared the clustering induced by *P. timonensis* and *M. elsdenii*, as these have previously been shown to activate DCs to a similar extent, suggesting that both strains have comparable amounts of LPS or other PAMPs. However, other bacteria can be tested to show that the effect is specific for *P. timonensis*. Moreover, it is possible that other bacteria are capable of inducing a similar clustering effect, and this might aid in the identification of potential shared underlying molecular mechanisms. Analyses of alternative adhesion pathways should elucidate which adhesion pathways are activated by *P. timonensis* to enhance clustering. Identifying the unique characteristics of *P. timonensis* involved in the induction of clustering of immune cells will be important for the design of targeted approaches to treat bacterial vaginosis and mucosal inflammation.

## 5 Materials and Methods

### 5.1 Ethics Statement

This study was performed in accordance with the ethical principles set out in the Declaration of Helsinki and was approved by the Medical Research Ethics Committee (MREC) of the Amsterdam University Medical Centers and the Ethics Advisory Board of Sanquin Blood Supply Foundation (Amsterdam, the Netherlands). Use of buffy coats is not subject to informed consent according to the Medical Research Involving Human Subjects Act (WMO) and MREC Amsterdam UMC. Human buffy coats from healthy volunteers were obtained after blood donation at Sanquin. All Buffy coat samples were processed anonymously and in compliance with the relevant guidelines and regulations outlined in the Amsterdam UMC Research Code.

### 5.2 Primary Cell Isolation and Cell Lines

CD14^+^ immature peripheral blood monocytes of healthy donors (Sanquin blood bank) were isolated by density gradient centrifugation on Lymphoprep (Axis‐Shield) and Percoll (Cytiva) as previously described [21, 22]. The isolated monocytes were differentiated into monocyte‐derived dendritic cells for six to seven days in the presence of IL‐4 (500 U/mL, Miltenyi Biotec) and GM‐CSF (800 U/mL, Miltenyi Biotec). CD4 T cells were isolated by magnetic labelling of peripheral blood mononuclear cells (PBMCs) using a human CD4 T cell isolation kit (Miltenyi Biotec) and consequent separation according to the manufacturer's instructions; cells were routinely >95% pure. For isolation of allogeneic memory CD4 T cells (characterized by expression of CD45RO), an additional labelling and separation was performed with anti‐CD45RO‐PE (Agilent) and anti‐PE‐microbeads (Miltenyi Biotec). DCs and CD4 T cells were maintained in Roswell Park Memorial Institute (RPMI) 1640 medium (Gibco/Life Technologies), supplemented with 10% heat‐inactivated fetal calf serum (FCS, Biological Industries), 2 mM L‐glutamine (Capricorn Scientific), 10 U/mL penicillin (Gibco/Life Technologies), and 10 µg/mL streptomycin (Gibco/Life Technologies). Four days after differentiation, DCs were seeded at 5 × 10^4^/well or 1 × 10^5^/well in 50 or 100 µL, respectively, in a 96‐well U‐bottom plate (Corning), and cultured for 2 days. DCs were exposed to microbiota at day five. For cell–cell clustering experiments, CD4 T cells were thawed and allowed to recover overnight in the presence of IL‐2 (20 U/mL, Miltenyi).

Monocytic THP‐1 (ATCC TIB‐202) and T‐lymphoid SupT1 (ATCC CRL‐1942) cell lines were maintained in complete RPMI1640 medium. All cultures were maintained at 37°C under 5% CO_2_ atmosphere.

### 5.3 Vaginal Bacteria

*P. timonensis* CRIS 5C‐B1 (alternative name *Hoylesella timonensis* [23], BEI Resources, HM‐136) and *M. elsdenii* (DSMZ‐20460) bacteria were cultured as recommended by DSMZ (German Collection of Microorganisms and Cell Cultures GmbH). Bacteria were harvested during logarithmic phase; the bacterial pellet was extensively washed with phosphate‐buffered saline (PBS), and culture purity was confirmed by Gram staining. The optical density at 600 nm (OD_600nm_) was measured, and bacterial suspensions were normalized to OD = 1 in PBS. Subsequently, bacteria were inactivated using a UV Crosslinker (Stratagene Stratalinker 1800 UV) by five rounds of exposure to 100,000 µJ/cm^2^ UV irradiation. Complete loss of bacterial viability was verified in liquid culture.

### 5.4 DC Integrin Activation Analysis

DCs were seeded at 5 × 10^4^/well and stimulated for 16 h with UV‐inactivated bacteria *P. timonensis* or *M. elsdenii* at a multiplicity of infection (MOI) of 10. As a positive control, cells were incubated with integrin stimulus phorbol 12‐myristate 13‐acetate (PMA, 100 ng/mL; Sigma) for 30 min at 37°C in TSA buffer (TSM (20 mM Tris, 150 mM NaCl, 1 mM CaCl2, 2 mM MgCl2) at pH 7.4 supplemented with 0.5% BSA). To assess activation of the LFA‐1 integrin receptor, cells were washed and stained with mAb anti‐human CD11a/CD18‐PE (1:25, clone m24, BioLegend) in TSA buffer for 30 min at 4°C. Cells were measured by flow cytometry using FACS Canto II (BD Biosciences). Data analysis of all flow cytometry data was performed using FlowJo v10.10.0 software (TreeStar), and the guidelines for the use of flow cytometry and cell sorting in immunological studies were adhered to [24]. Live cells were gated using FSC and SSC, single cells were gated using SSC‐A vs SSC‐H, and the geometric mean of the fluorescent intensity (MFI) of LFA‐1 was determined.

### 5.5 Cell–Cell Clustering Assays and Imaging Flow Cytometry

To determine cell–cell clustering, DCs or THP‐1 cells (1 × 10^6^ cells/mL) were fluorescently labelled with 1:5000 CellTrace Carboxyfluorescein Succinimidyl Ester (CFSE) proliferation kit (ThermoFisher Scientific) for 10 min at 37°C while shaking. To remove any free dye, cells were washed three times with PBS. CFSE‐labelled cells were seeded (1 × 10^4^/well) and exposed for 16 h to *P. timonensis* or *M. elsdenii* (MOI 10). 1 × 10^6^ cells/mL of THP‐1, SupT1, or primary CD4 T cells were labelled with 1:1000 dihydroethidium (HE, ThermoFisher Scientific) for 30 min at 37°C while shaking and subsequently washed three times with PBS. CFSE‐labelled cells were mixed with HE‐labelled cells in a 1:1 ratio and co‐cultured for up to 120 min in medium at 37°C, with measurements taken every 30 min. Cell–cell clustering was determined by flow cytometry using FACS Canto II. Double positive cells (HE/CFSE) within the live gate (FSC‐A/SSC‐A) were considered as clustered cells.

Alternatively, to visualize and quantify cell–cell clustering by imaging flow cytometry, 10 × 10^6^ cells/mL of SupT1 cells were fluorescently labelled with 0.5 µM CellTrace Violet (CTV) Cell Proliferation Kit (ThermoFisher Scientific) according to the manufacturer's instructions. CFSE‐labelled DCs were mixed with CTV‐labelled SupT1 cells in a 1:1 ratio, and clustering was measured over time from 0 to 900 s at 40× magnification by imaging flow cytometry (Amnis ImageStream^X^ Mark II, Cytek Biosciences). Analysis was performed using IDEAS v6.2 software (Amnis). Cells in focus of the flow system were gated (normalised frequency vs. gradient RMS in the Brightfield channel), followed by cells in a specific time frame (0–30 s, 30–90 s, 90–150 s, 150–300 s, 300–450 s, 450–600 s, 600–900 s) (intensity DC CFSE vs. time). Next, gates were set to determine single CFSE‐positive cells (DC), single CTV‐positive cells (T), and double‐positive cells (T + DC), the latter comprising the clustered cells (intensity DC CFSE vs. intensity SupT1 CTV) (Figure S1). Cell–cell clustering (T + DC) was depicted as a percentage of all CFSE‐positive cells. To determine spectral overlap, single‐stained samples were acquired for each fluorescent channel, for the different DC stimulations, and for SupT1 cells.

### 5.6 ICAM‐1 Bead Binding Assay

PerCP fluorescent beads (TransFluoSpheres, Molecular Probes) were covalently crosslinked to streptavidin, and subsequently incubated with biotinylated goat‐anti‐human anti‐Fc‐specific F(ab’)_2_ and ICAM‐1‐Fc (provided by Prof. Yvette van Kooyk) as previously described [14]. 5 × 10^4^/well were seeded and stimulated for 16 h with UV‐inactivated *P. timonensis* or *M. elsdenii* (MOI 10). Cells were transferred to a 96‐well V‐bottom plate (Starstedt) and exposed to TSA in the presence or absence of blocking antibody anti‐LFA‐1 (40 µg/mL, NKI‐L15, provided by Prof. Yvette van Kooyk) for 30 min at 37°C in TSA. Subsequently, DCs were incubated with ICAM‐1 beads for 30 min at 37°C. Cells were washed, resuspended in TSA, and bead binding was assessed by flow cytometry using FACS Canto II. Live cells were gated using FSC and SSC, and the percentage of cells that had bound ICAM‐1 beads was determined.

### 5.7 T Cell Proliferation Assay

To determine T cell proliferation, DCs were seeded at 5 × 10^4^/well and exposed for 16 h to *P. timonensis* or *M. elsdenii* (MOI 10). Allogeneic memory CD4 T cells were loaded with 2 µM CFSE for 10 min at 37°C and washed. Cells were counted and DCs (1 × 10^4^/well) were co‐cultured with CFSE‐labelled memory CD4 T cells (4 × 10^4^/well) for five days in a 96‐well F‐bottom plate (Corning) at 37°C. To assess maximum proliferation, untreated DCs were co‐cultured with memory CD4 T cells in the presence of phytohemagglutinin (PHA, 1 µg/mL, Welcome) and IL‐2 (50 U/mL). At day five, memory CD4 T cells were harvested and stained for viability using Fixable Viability Dye eFluor 780 (1:4000, eBioscience) in PBS for 10 min at 4°C. T cell proliferation was assessed as loss of CFSE label in viable cells by FACS Canto II. Cells staining positive for the live–dead marker (Fixable Viability Dye eFluor 780) were excluded from analysis. The division index of memory CD4 T cells was determined using the proliferation tool in FlowJo and depicts the total number of cell divisions/the total number of viable, CFSE‐labelled cells at the start of culture.

### 5.8 Data Analysis and Statistics

Generation of graphs and statistical analyses were performed using GraphPad Prism v10.2.0 software (GraphPad Software Inc.). A two‐way ANOVA test was performed for multiple comparisons of unpaired grouped data between donors with a Tukey's multiple comparisons test. Results are presented as mean +SD. Statistical significance was set at *p* < 0.05 (ns = not significant, *p < 0.05; **p < 0.01, ***p < 0.001, ****p < 0.0001).

## Author Contributions

Marleen Y. van Smoorenburg designed experiments. Marleen Y. van Smoorenburg, John L. van Hamme, Julia L. Nerwinska, and Ester B. M. Remmerswaal performed the experiments. Celia Segui‐Perez and Karin Strijbis contributed essential research materials and scientific input. Marleen Y. van Smoorenburg, Julia L. Nerwinska, Ester B. M. Remmerswaal, and Teunis B. H. Geijtenbeek analyzed and interpreted data. Marleen Y. van Smoorenburg and Teunis B. H. Geijtenbeek wrote the manuscript with input from all listed authors. Teunis B. H. Geijtenbeek supervised all aspects of the study.

## Conflicts of Interest

All authors declare no conflicts of interest.

## Peer Review

The peer review history for this article is available at https://publons.com/publon/10.1002/eji.70051.

## Supporting information




**Supporting Information file 1**: eji70051‐sup‐0001‐SuppMat.pdf

## Data Availability

Data are available from the corresponding author upon reasonable request.
