# Supplementary material for: Bacterial Vaginosis‐Associated Prevotella timonensis Enhances Dendritic Cell–T Cell Clustering and Subsequent T Cell Proliferation
Source: Eur J Immunol. 2025 Sep 3;55(9):e70051. doi: 10.1002/eji.70051 (PMC12405971; doi:10.1002/eji.70051)
Supplement: Supplementary file 1 — Supporting Information file 1: eji70051‐sup‐0001‐SuppMat.pdf [file EJI-55-e70051-s001.pdf]

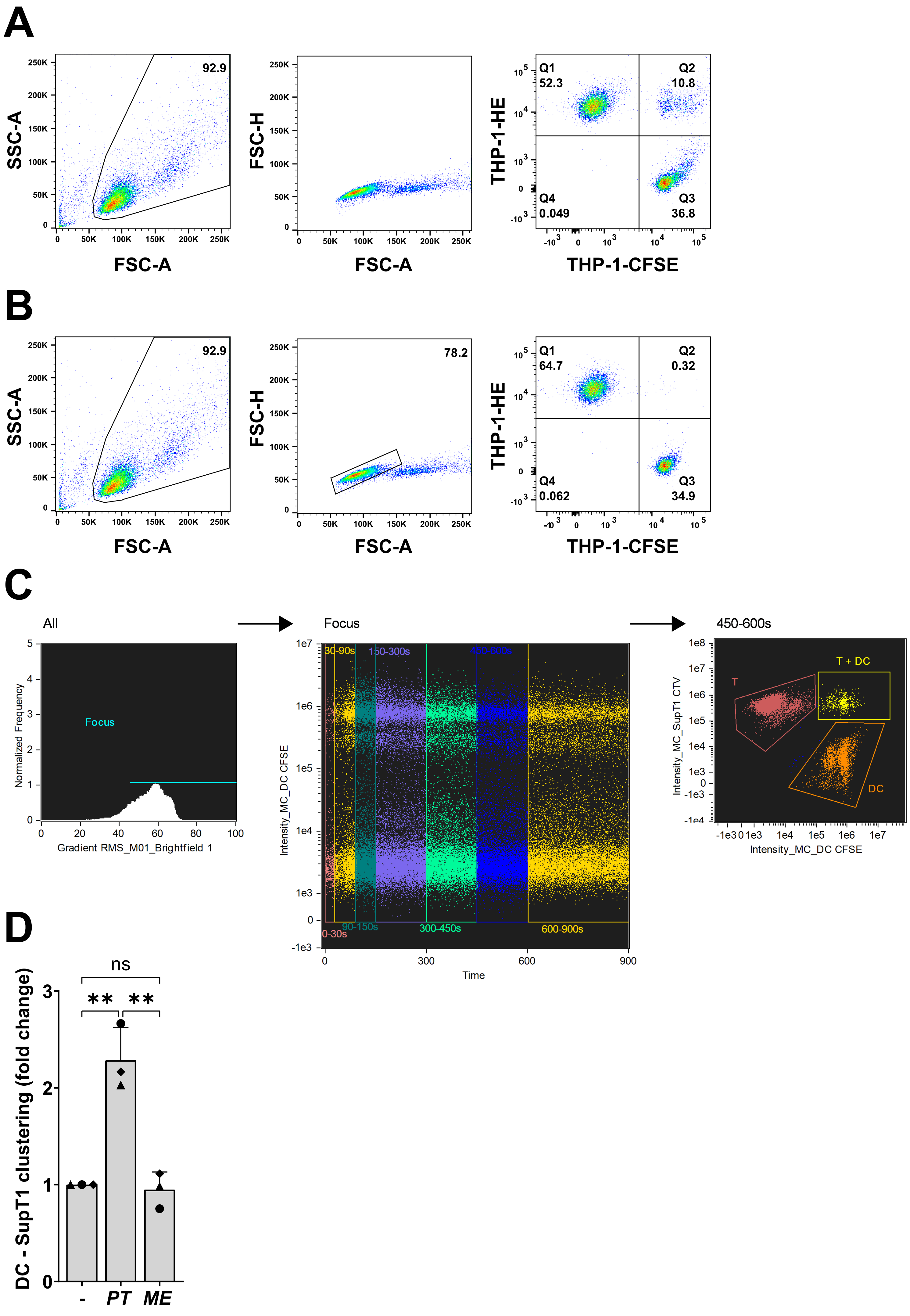

A
SSC-A
FSC-A
92.9
FSC-H
FSC-A
THP-1-HE
THP-1-CFSE
Q1
52.3
Q2
10.8
Q3
36.8
Q4
0.049
B
SSC-A
FSC-A
92.9
FSC-H
FSC-A
78.2
THP-1-HE
THP-1-CFSE
Q1
64.7
Q2
0.32
Q3
34.9
Q4
0.062
C
All
Focus
Normalized Frequency
Gradient RMS_M01_Brightfield 1
Focus
Intensity_MC_DC CFSE
Time
0-30s
30-90s
90-150s
150-300s
300-450s
450-600s
600-900s
450-600s
Intensity_MC_SupT1 CTV
Intensity_MC_DC CFSE
T
T + DC
DC
D
DC - SupT1 clustering (fold change)
ns
**
**
-
PT
ME

Figure S1. Gating strategies (imaging) flow cytometry

Carboxyfluorescein Succinimidyl Ester (CFSE; 1:5000)-labelled THP-1 cells (**A, B**) or DCs (**C, D**) were stimulated with UV-inactivated bacteria for 16 h (MOI 10) and co-cultured with dihydroethidium (HE; 1:1000)-labelled THP-1 cells for 2 h in a 1:1 ratio. **A-B**. Clustering was measured every 30 minutes by flow cytometry. Gating strategy for a representative experiment, depicting *P. timonensis*-exposed THP-1 cells at 1 h excluding (**A.**) or including (**B.**) a single cell gate (FSC-A vs FSC-H). For all clustering-related graphs the upper gating strategy excluding a single cell gate (**A.**) was used. Live cells were gated based on FSC-A vs SSC-A and HE/CFSE double positive events (Q2) were considered clustered cells. **C-D**. CFSE-labelled DCs were co-cultured with CellTrace Violet (CTV)-labelled SupT1 cells in a 1:1 ratio and clustering was measured for 900 seconds by imaging flow cytometry. **C.** An example of the used gating strategy depicting a sample of *P. timonensis*-exposed DCs with SupT1 cells. Cells in focus of the flow system were gated (Normalised Frequency versus Gradient RMS in the Brightfield channel), followed by time gates (Intensity DC CFSE versus Time) to assess cell-cell clustering over time. In each time gate, the same gates were set to determine single CFSE positive cells (DC), single CTV positive cells (T) and double positive cells (T + DC), the latter comprising the clustered cells (Intensity DC CFSE versus Intensity SupT1 CTV). **D.** Cell-cell clustering (T + DC gate) was calculated as a percentage of all CFSE positive cells and depicted as fold change compared with cell-cell clustering in untreated DCs. Symbols represent three independent donors, bars represent mean +SD. $^{**}P < 0.01$.
